# Support for Chronic Pain Management for Breast Cancer Survivors Through Novel Digital Health Ecosystems: Pilot Usability Study of the PainRELife Mobile App

**DOI:** 10.2196/51021

**Published:** 2024-02-02

**Authors:** Marianna Masiero, Chiara Filipponi, Elisa Fragale, Silvia Francesca Maria Pizzoli, Elisabetta Munzone, Alessandra Milani, Luca Guido, Vittorio Guardamagna, Sara Marceglia, Roberto Prandin, Marco Prenassi, Annamaria Caruso, Vania Manzelli, Chiara Savino, Costanza Conti, Federica Rizzi, Alice Casalino, Giulia Candiani, Francesca Memini, Luca Chiveri, Andrea Luigi Vitali, Massimo Corbo, Roberto Grasso, Florence Didier, Roberta Ferrucci, Gabriella Pravettoni

**Affiliations:** 1 Department of Oncology and Hemato-Oncology University of Milan Milan Italy; 2 Applied Research Division for Cognitive and Psychological Science European Institute of Oncology Istituto di Ricovero e Cura a Carattere Scientifico Milan Italy; 3 Division of Medical Senology European Institute of Oncology Istituto di Ricovero e Cura a Carattere Scientifico Milan Italy; 4 Nursing School European Institute of Oncology Istituto di Ricovero e Cura a Carattere Scientifico Milan Italy; 5 Division of Palliative Care and Pain Therapy European Institute of Oncology Istituto di Ricovero e Cura a Carattere Scientifico Milan Italy; 6 Dipartimento di Ingegneria e Architettura Università degli Studi di Trieste Milan Italy; 7 Nuvyta, Società a Responsabilità Limitata Cologno Monzese Italy; 8 Istituto di Management Sanitario Milano Italy; 9 Agenzia di comunicazione scientifica Zadig, Società a Responsabilità Limitata, Società benefit Milan Italy; 10 Dipartimento di Scienze Neuroriabilitative Casa di Cura del Policlinico Milan Italy

**Keywords:** chronic pain, eHealth, cancer, decision-making, survivorship, self-efficacy, pain, oncology, health ecosystem, health ecosystems, breast, survivor, survivors, mHealth, mobile health, app, apps, applications, MARS

## Abstract

**Background:**

Chronic pain is one of the most common and critical long-term effects of breast cancer. Digital health technologies enhance the management of chronic pain by monitoring physical and psychological health status and supporting pain self-management and patient treatment decisions throughout the clinical pathway.

**Objective:**

This pilot study aims to evaluate patients’ experiences, including usability, with a novel digital integrated health ecosystem for chronic pain named PainRELife. The sample included patients with breast cancer during survivorship. The PainRELife ecosystem comprises a cloud technology platform interconnected with electronic health records and patients' devices to gather integrated health care data.

**Methods:**

We enrolled 25 patients with breast cancer (mean age 47.12 years) experiencing pain. They were instructed to use the PainRELife mobile app for 3 months consecutively. The Mobile Application Rating Scale (MARS) was used to evaluate usability. Furthermore, pain self-efficacy and participation in treatment decisions were evaluated. The study received ethical approval (R1597/21-IEO 1701) from the Ethical Committee of the European Institute of Oncology.

**Results:**

The MARS subscale scores were medium to high (range: 3.31-4.18), and the total app quality score was 3.90. Patients with breast cancer reported reduced pain intensity at 3 months, from a mean of 5 at T0 to a mean of 3.72 at T2 (*P*=.04). The total number of times the app was accessed was positively correlated with pain intensity at 3 months (*P*=.03). The engagement (*P*=.03), information (*P*=.04), and subjective quality (*P*=.007) subscales were positively correlated with shared decision-making. Furthermore, participants with a lower pain self-efficacy at T2 (mean 40.83) used the mobile app more than participants with a higher pain self-efficacy (mean 48.46; *P*=.057).

**Conclusions:**

The data collected in this study highlight that digital health technologies, when developed using a patient-driven approach, might be valuable tools for increasing participation in clinical care by patients with breast cancer, permitting them to achieve a series of key clinical outcomes and improving quality of life. Digital integrated health ecosystems might be important tools for improving ongoing monitoring of physical status, psychological burden, and socioeconomic issues during the cancer survivorship trajectory.

**International Registered Report Identifier (IRRID):**

RR2-10.2196/41216

## Introduction

In 2040, it is expected there will be approximately 26 million new cancer survivors in the United States, underscoring the growing importance of survivorship [1,2]. Mullan [3] defined survivorship as a process characterized by 3 different stages: acute survival, from diagnosis to active treatments; extended survival, from treatments to active surveillance; and permanent survival, in which the probability of recurrence is low. As suggested by Mullan’s definition, cancer survivorship is a complex, multistep, dynamic process characterized by evolving needs and challenges. Pain, fatigue, and psychological distress (eg, anxiety, depression, worry, and fear of cancer recurrence) are typical long-term effects that deleteriously affect survivors’ engagement in work, personal, and social activities [4-6]. In particular, one of the most common and critical long-term effects of cancer in survivors is chronic pain. It has been linked with several physical, psychological, and socioeconomic sequelae. A study by Jiang et al [1] stated that, of 4526 cancer survivors, around 34.6% reported chronic pain, and 16.1% conveyed that it limited daily life and work activities. Notwithstanding, chronic pain throughout the survivorship trajectory is underinvestigated and undertreated [5,7,8]. Since chronic pain affects the quality of life (QoL) of patients with breast cancer [9], it is essential to design, test, and implement patient-driven interventions [10] that enable ongoing monitoring of physical and psychological health status, from the “hospital to the patient’s home,” and support pain self-management and patient treatment decisions throughout the entire clinical pathway. This might be particularly important in extended and permanent survivorship, reducing the risk of cancer survivors exiting the care system [5].

A growing body of evidence has shown that using digital health technologies integrated into dynamic ecosystems enhances the management of chronic pain by assessing patients' physical health and psychological well-being and providing tailored interventions [11]. Digital health technologies aim to integrate various digital tools and technologies, including patient electronic health records, telemedicine, wearables, and mobile apps, into the health care system [12]. Overall, digital health technology encompasses both eHealth, which involves the use of the internet and related technologies to enhance health care systems through information and communication technology [13,14], and mobile health, which focuses on health practices supported by mobile devices [15]. The widespread use of digital health technologies has opened an innovative “window of opportunity” for managing chronic pain in a more personalized, accessible, and patient-centered way [8]. Evidence has highlighted that digital health technologies are valuable solutions for remotely collecting patient health data (eg, using patient-reported measures or wearable devices), improving symptom management, and decreasing patient appointments and hospitalizations [16]. Jongerius et al [17], in a systematic review, highlighted that digital health technologies are used in cancer clinical practice for the following different reasons: to stimulate the adoption of preventive behaviors, to increase early cancer identification, to manage cancer care, and to provide assistance to cancer survivors. Overall, digital health technology supports patients and the health care system to achieve several critical outcomes for better cancer clinical management [17-19]. For example, Zhu et al [19] reported that an app-based program named “Breast Cancer e-Support” was able to decrease symptomologies related to anticancer treatments and therefore improve self-efficacy and QoL. In addition, Maguire et al [20] designed and tested ASyMS (Advanced Symptom Management System) for the management of chemotherapy toxicity; it not only enables the evaluation and monitoring of patient symptomatology related to anticancer treatments but also provides tailored and evidence-based recommendations to manage symptoms [20].

Considering the specific case study of chronic pain in cancer survivorship, digital health technologies enhance access to nonpharmacological interventions; address pain-related mobility issues; improve patient networking and connections; foster self-management, self-efficacy, coping, and patient engagement; and facilitate communication among health care professionals of various specialties (eg, physicians, nurses, physiotherapists, psychologists) [18,21,22]. For example, Ranney et al [18] described that 89.5% of patients with chronic pain reported using digital health tools (eg, websites to search for health information, social media, and mobile apps), and such usage is associated with improved chronic pain coping mechanisms*.*

Different digital health technologies have been designed to manage cancer and pain in the clinical pathway [23]. For example, an educational digital intervention called the “Pain Education after Cancer Collaborative” (PECAN project) was developed for survivors of breast cancer; in the intervention, a decision tree system is used to provide tailored educational information to cancer survivors based on their answers to specific queries [24]. In addition, a Mobile Pain Coping Skills Training Protocol has been proposed to support patients’ understanding of the pain experience and strategies to cope with the pain [25]. More generally, Hauser-Ulrich et al [26] recently developed a text-based chatbot named “SELMA” (PainSELfMAnagement) aimed to booster self-management of chronic pain in different types of diseases, supporting health care professionals in the delivery of evidence-based interventions. Moreover, digital health technologies could encourage patients to be more involved in their treatment decisions, through the implementation of specific decision aids [16,27-29]. Studies have stressed that the implementation of tailored decision aids in mobile apps increases patients’ awareness about treatment preferences (eg, pharmacological vs nonpharmacological treatments), reduces decisional conflict, and enhances adherence to treatments [30-32].

Even with the key role of digital health technologies in the cancer clinical pathway, few studies on digital and integrated health ecosystems are currently available [33]. Consequently, in this pilot study, our primary endpoint was the usability of the novel digital integrated health ecosystem, PainRELife, for managing and monitoring chronic pain in patients with breast cancer throughout the survivorship trajectory. Further, we aimed to evaluate the PainRELife ecosystem's effectiveness at enhancing pain self-efficacy, improving shared decision-making, and reducing pain perception as secondary endpoints. The PainRELife ecosystem stands out as the first to seamlessly integrate all the essential components required for comprehensive pain management within a single platform. This includes features such as pain monitoring, physical and psychological assessment, e-diaries, exercises, educational resources, and decision aids. Furthermore, it incorporates a dedicated platform for health care providers and a robust big data cloud infrastructure. This holistic integration sets our ecosystem apart in the realm of pain management solutions.

## Methods

### Study Design and Patient Recruitment

#### PainRELife Ecosystem

**Figure 1:**
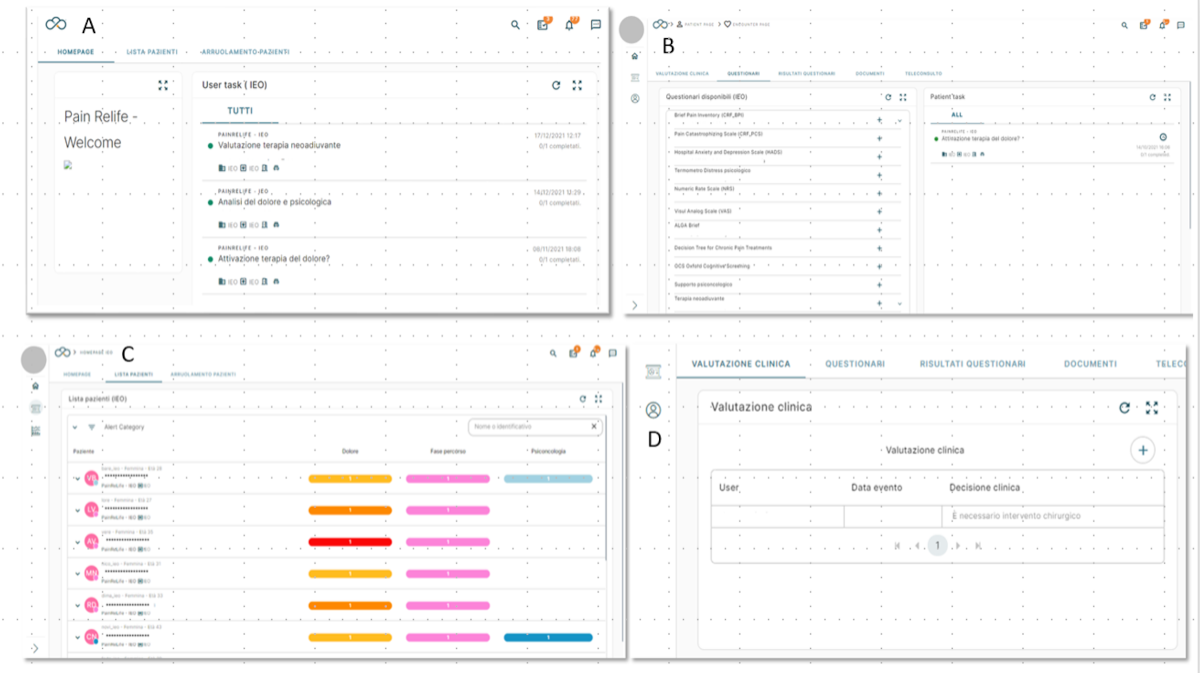
Health care professional interface on the Nu Platform: (A) home page displaying all available activities for health care professionals, (B) patient questionnaire list providing the measures used to assess psychological and physical status administered via the PainRELife mobile app, (C) patient list providing a directory of all patients registered on the Nu Platform, (D) clinical evaluation page offering access to detailed information on clinical events and therapeutic suggestions.

This pilot study is nested in a national project titled *“*PainRELife, Sustainable and integrated big data ecosystem for continuity of care and decision support for patients with pain“ (ID: 1173269). This national project guided the development and testing of an integrated health ecosystem for the management of chronic pain. Specifically, the PainRELife ecosystem consists of a cloud technology platform interconnected with electronic health records, which is named the Nu Platform, connected to the Fast Healthcare Interoperability Resources (FHIR) server for data analysis related to the patient care pathway. Health care providers use the Nu Platform to collect and store patient clinical data, and it enables the ongoing monitoring of patient health status (eg, pain, psychological well-being, and decision preferences about treatments; see Figures 1A and 1B), from diagnosis and active treatments to follow-ups (see Figures 1C and 1D). In addition, a big data infrastructure linked to the FHIR server enables a series of dynamic dashboards aimed at providing a systematic, intuitive outline of patient population features that might be used by researchers, clinicians, and health care stakeholders. The Nu Platform is associated with a mobile app for patients named PainRELife, which collects health care data. This technological solution permits dual communication between patients and health care professionals. Information collected by the mobile app is saved in the Nu Platform and overseen by health care professionals [7].

**Figure 2:**
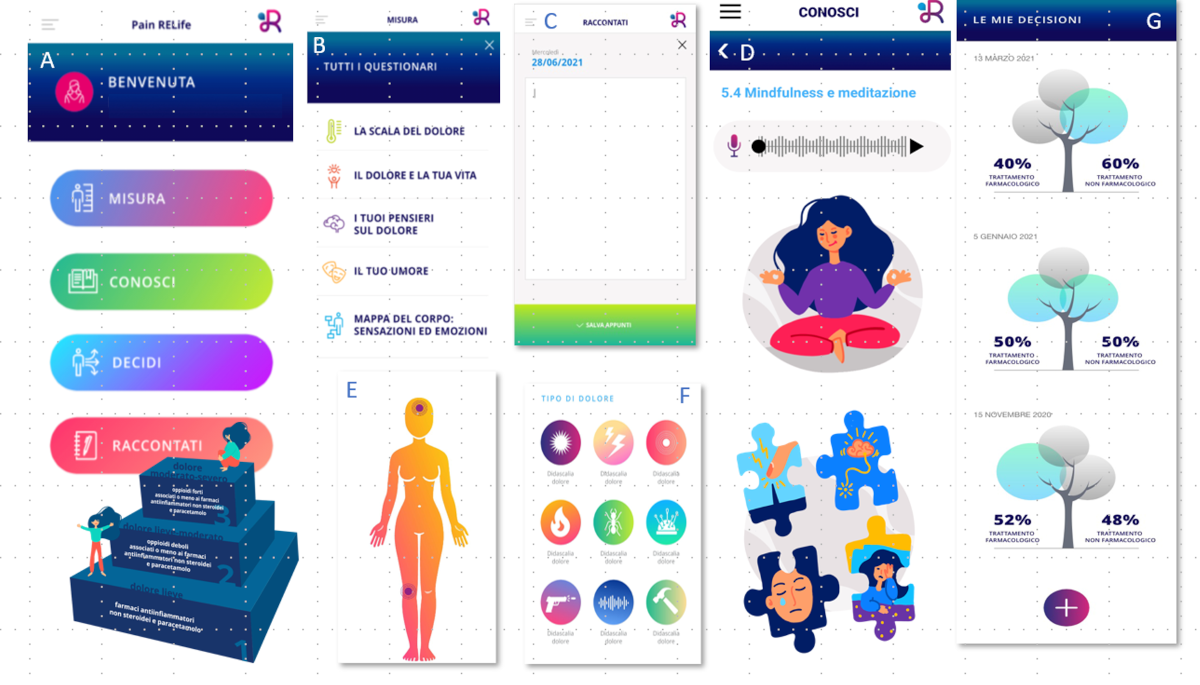
Patient interface on the PainRELife mobile app: (A) home page showing an overview of the mobile app sections, (B) pain and psychological well-being assessment section displaying the questionnaires that patients are required to complete, (C) patient’s e-diary, (D) educational section showing some of the educational content within the mobile app, (E) and (F) pain and emotion-body mapping exercises, (G) decision aid section showing an example for preferences for pharmacological and nonpharmacological treatments.

The PainRELife mobile app enables patient education and the collection of patient-reported outcomes. The mobile app is composed of an ”educational section“ that includes educational resources to improve learning about chronic pain throughout the cancer pathway (throughout the different phases of survivorship: acute, extended, and permanent; see Figure 2D) [3] and a “pain and psychological well-being assessment section” that contains a set of validated questionnaires (eg, pain intensity and interference, anxiety, and depression; see Figures 2A and 2B). Furthermore, the mobile app includes an e-diary (see Figure 2C) and exercises for pain and emotion-body mapping (see Figures 2E and 2F), enabling a holistic evaluation of psychological well-being and the pain experience. In addition, the mobile app incorporates a decision aid section, which is structured in 2 modules: profiling patients’ preferences and a decision tree (see Figure 2G). These modules are designed to empower patients with breast cancer by increasing their awareness of treatment preferences and facilitating shared decision-making regarding their care. The “profiling patients’ preferences” module aims to assist patients with evaluating and comprehending essential aspects of both pharmacological and nonpharmacological treatments. This includes understanding how interventions and treatments work to reduce pain or aid in recovery, identifying their advantages, and recognizing potential disadvantages. The decision tree module enables patients with breast cancer to tailor their health care preferences using the subjective expected utility approach [7] (see Figure 2G). This empowers patients with breast cancer to actively participate in the decision-making process, aligning their treatment choices with their unique needs and goals.

#### Participants

Participants of this pilot usability study included 25 patients with diagnosed breast cancer and pain (mean age 47.12, SD 8.41 years) admitted to the Division of Medical Senology and the Division of Pain Therapy and Palliative Care of the European Institute of Oncology (IEO). Participants were introduced to the mobile app after their clinic visit and instructed to use it for 3 months consecutively. Participants were recruited according to the following established set of inclusion criteria: >18 years old, affected by breast cancer, has undergone surgical intervention for breast carcinoma, experiencing post-surgical pain (≥3 on a numeric rating scale [NRS]), and in possession of internet access and a personal smartphone. We excluded patients with breast cancer who had a previous or ongoing psychiatric or neurological disorder or other disease requiring active analgesic treatments. The inclusion and exclusion criteria were established considering that chronic pain is a common side effect (related to both the surgery and anticancer treatments) for patients with breast cancer (~60% experience it), and persistent acute pain after surgery is considered a risk factor for developing chronic pain during survivorship [34]. A full and detailed description of the research protocol of this pilot study was published previously [7].

#### Instruments

Patient sociodemographic and medical data were gathered through electronic medical records and a set of ad hoc items during the enrollment consultation. For the perceived pain assessment, the NRS was used to evaluate pain using a numerical range from 0 (no pain) to 10 (severe pain) [35]. Further, a set of validated self-measures was used to evaluate the primary and secondary endpoints. In detail, the Mobile Application Rating Scale (MARS) was used to evaluate the eHealth platform usability. MARS is a self-administered questionnaire with 29 items evaluating the following dimensions: engagement; functionality; aesthetics; quality of the information received; subjective perception of the app quality; impact of the mobile app on knowledge, attitudes, and probability of modifying the target individual behaviors (in this specific case, it refers to behaviors related to pain management). The Cronbach α of the NRS is .90, indicating good internal consistency [36-38]. The Pain Self-Efficacy Questionnaire (PSEQ) is a self-administered questionnaire consisting of 10 items that evaluate self-efficacy in patients with pain. The Cronbach α is .94, indicating excellent internal consistency [39,40]. Finally, the 9-item Shared Decision-Making Questionnaire (SDM-Q-9) is a self-administered questionnaire comprising 9 items that evaluate a series of aspects related to the possibility of achieving a shared decision [41]. The Cronbach α is .94, indicating excellent internal consistency [42].

### Statistical Considerations

A series of descriptive analyses were performed to depict the characteristics of the sample. In order to evaluate the primary endpoint, the mean score and its SD were calculated for each MARS subscale (engagement experienced while using the app; functionality; aesthetics; quality of the information received; subjective perception of the app quality; expected impact on knowledge, attitudes, and probability to change user behaviors) at 3 months; in addition, the total number of times the PainRELife mobile app was accessed by each participant was determined.

Further, a new variable named total app quality was created using the mean values of the engagement, functionality, aesthetics, and information quality MARS subscales. The final measurement of app quality was the average value of the 4 means [43]. Pearson correlational analysis was performed among all self-reports used (NRS, PSEQ, SDM-Q-9, MARS) and the total number of times the app was accessed during the 3 months of the study. A repeated measures ANOVA was performed to detect variation in pain intensity (NRS) from T0 (baseline) to T2 (3 months). Further, a new dichotomous variable named “frequency of use” was created considering the entire number of times the app was accessed (mean 22.92, SD 15.60; range 2-73) and the lowest number of times the PainRELife mobile app needed to be accessed (21 times) by participants to finalize the study’s tasks. The “frequency of use” variable permitted dividing the participants into groups based on higher and lower frequencies of access. Further, a Student *t* test was run to evaluate the difference between the “frequency of use” and PSEQ, SDM-Q-9, and MARS scores. Data were analyzed using SPSS version 26.0 (IBM Corp). 

### Ethical Considerations

This study received ethical approval in December 2021 (R1597/21-IEO 1701) from the Ethical Committee of the IEO and respects the Declaration of Helsinki and Good Clinical Practice Guidelines. All participants read and signed the informed consent form, which encompassed a comprehensive and exhaustive explanation of the primary and secondary endpoints, study procedures, and possible risks and benefits. Participants were not compensated and were able to withdraw their participation at any time during the study. Concerning privacy and confidentiality protection, all data collected were deidentified and anonymized, complied with national data protection legislation, and will be stored in the IEO databases for 10 years.

The authors affirm that human research participants provided informed consent for publication of their data.

## Results

### Participant Characteristics

The sociodemographic, cancer, and treatment characteristics are listed in Tables 1-3.

**Table 1:** Sociodemographic information of participating patients with breast cancer (n=25).

Characteristics			Results, n (%)
**Marital status**			
	Cohabiting	1 (4)	
	Widowed	3 (12)	
	Single	5 (20)	
	Married	16 (64)	
**Educational level**			
	PhD	2 (8)	
	Master’s degree	8 (32)	
	High school	12 (48)	
	Primary school	3 (12)	

**Table 2:** Diagnosis, cancer type, familiarity, and genetic mutation of participating patients with breast cancer (n=25).

Cancer characteristics			Results, n (%)
**Diagnosis**			
	Lobular carcinoma	3 (12)	
	Ductal carcinoma	17 (68)	
	Ductal carcinoma in situ	3 (12)	
	Mucinous carcinoma	1 (4)	
	Occult carcinoma	1 (4)	
**Cancer type**			
	Triple negative	2 (8)	
	HER2+^a^	5 (20)	
	Luminal	18 (72)	
**Familiarity**			
	I° breast	8 (32)	
	II° breast	6 (24)	
	No familiarity	11 (44)	
**Mutation**			
	BRCA1	2 (8)	
	BRCA1	2 (8)	
	Negative	6 (24)	
	No testing	13 (52)	

^a^HER2: human epidermal growth factor receptor 2.

**Table 3:** Surgery, medical treatments, and radiotherapy undergone by participating patients with breast cancer (n=25).

Treatment characteristics			Results, n (%)
**Surgery**			
	Mastectomy	23 (92)	
	Axillary dissection	1 (4)	
	Quadrantectomy	1 (4)	
**Medical treatment**			
	Chemotherapy + endocrine therapy	8 (32)	
	Chemotherapy + immune therapy	2 (8)	
	Endocrine therapy	12 (48)	
	Immune + endocrine therapy	1 (4)	
	No treatment	2 (8)	
**Radiotherapy**			
	Yes	8 (32)	
	No	17 (68)	

### Usability

The total MARS score (ranging from 1 to 5) provided overall medium-to-high mean values for each subscale (range 3.31-4.18; see Table 4) and a mean total app quality score of 3.90 (SD 0.506), suggesting generally good usability as evaluated by the participants. This was also confirmed by the total number of times the participants accessed the app during the entire study (mean 22.92, SD 15.60; range 2-73). In particular, 3 of 5 subscales had the highest scores: functionality (mean 4.14, SD 0.630), information (mean 4.18, SD 0.608), and behavioral change (mean 4.05, SD 0.666).

On the functionality subscale, 57% (15/23) of the participants judged that the mobile app is straightforward to use. Moreover, 91% (21/23) of the participants affirmed that the interactions are reliable and intuitive (ease of use: 8/23, 35% agree; 13/23, 57% strongly agree), positively evaluated the design (gestural design: 8/23, 35% agree; 12/23, 52% strongly agree), and evaluated the navigation properties as good (navigation: 12/23, 52% agree; 8/23, 35% strongly agree). However, some slight uncertainties were observed regarding the general performance of the mobile app, specifically moving between pages and sections (performance: 8/23, 35% undecided; see Table 5).

Concerning the distribution of responses in the information subscale, 78% (18/23) of the participants reported that the information in the mobile app is evidence-based (information: 9/23, 39% agree; 9/23, 39% strongly agree), relevant, focused on chronic pain in breast cancer and its management during the disease clinical pathway (quality of information: 9/23, 39% agree; 11/23, 48% strongly agree), and trustworthy (credibility: 22/23, 96% strongly agree). Further, the amount of information (quantity of information: 7/23, 30% agree; 9/23, 39% strongly agree) and how the information is reported using different setups (visual information: 11/23, 48% agree; 9/23, 39% strongly agree) were considered positive by the participants (see Table 6). Finally, most participants stated that the mobile app's goals are achievable (goals: 11/23, 48% agree; 3/23, 13% strongly agree), even if 30% (7/23) reported some concerns.

Last, the distribution of responses in the behavioral change subscale revealed that 83% (19/23) of the participants strongly agreed that the mobile app had improved awareness about the issue of chronic pain in the cancer disease pathway, and 70% (16/23) agreed that the app increased chronic pain–related knowledge. Likewise, 70% (16/23) of the participants reported that the mobile app might support attitudes toward chronic pain (attitudes: 9/23, 39% agree; 7/23, 30% strongly agree; see Table 7).

In addition, most of the participants reported that the mobile app would potentially be helpful to bolster help-seeking behaviors (help seeking: 5/23, 22% agree; 9/23, 39% strongly agree) as well as support an intention to change (intention to change: 5/23, 22% agree; 9/23, 39% strongly agree). Still, 39% (9/23) showed concerns about the capacity to transform intention into a fundamental behavioral change (see Table 7). Overall, participants judged the app to be well-targeted (engagement subscale: mean 3.31, SD 0.617) and the app’s layout to be adequate (aesthetics subscale: mean 3.98, SD 0.849); likewise, the overall subjective quality was adequate (subjective quality subscale: mean 3.50, SD 0.494).

**Table 4:** Mobile Application Rating Scale (MARS) subscale scores.

MARS subscales	Results, mean (SD)
Engagement	3.31 (0.617)
Functionality	4.14 (0.630)
Aesthetics	3.98 (0.850)
Information	4.18 (0.608)
Subjective quality	3.50 (0.494)
Behavioral change	4.05 (0.666)
Total app quality	3.90 (0.506)

**Table 5:** Functionality assessment of the PainRELife mobile app using the Mobile Application Rating Scale (MARS) [37,38] (n=23).

Functionality assessment	Response, n (%)				
	Strongly disagree	Disagree	Undecided	Agree	Strongly agree
Ease of use^a^	0	0	2 (9)	8 (35)	13 (57)
Gestural design^b^	1 (4)	0	2 (9)	8 (35)	12 (52)
Navigation^c^	0	1 (4)	2 (9)	12 (52)	8 (35)
Performance^d^	0	3 (13)	8 (35)	7 (30)	5 (22)

^a^“How easy is it to learn how to use the app?”; “How clear are the menu labels/icons and instructions?”

^b^“Are interactions (taps/swipes/pinches/scrolls) consistent and intuitive across all components/screens?”

^c^“Is moving between screens logical/accurate/appropriate/uninterrupted; are all necessary screen links present?”

^d^“How accurately/fast do the app features (functions) and components (buttons/menus) work?”

**Table 6:** Information assessment of the PainRELife mobile app using the Mobile Application Rating Scale (MARS) [37,38] (n=23).

Information assessment	Response, n (%)				
	Strongly disagree	Disagree	Undecided	Agree	Strongly agree
Information^a^	0	0	5 (22)	9 (39)	9 (39)
Credibility^b^	0	0	0	1 (4)	22 (96)
Quality of information^c^	1 (4)	0	2 (9)	9 (39)	11 (48)
Quantity of information^d^	1 (4)	0	6 (26)	7 (30)	9 (39)
Visual information^e^	0	2 (9)	1 (4)	11 (48)	9 (39)
Goals^f^	2 (9)	0	7 (30)	11 (48)	3 (13)

^a^”Contains high-quality information (eg, text, feedback, measures, references) from a credible source.“

^b^“Does the app come from a legitimate source (specified in app store description or within the app itself)?”

^c^“Is app content correct, well written, and relevant to the goal/topic of the app?”

^d^”Is the extent of coverage within the scope of the app and comprehensive but concise?”

^e^“Is the visual explanation of concepts—through charts/graphs/images/videos, etc—clear, logical, correct?”

^f^”Does app have specific, measurable, and achievable goals (specified in the app store description or within the app itself)?”

**Table 7:** Behavior change assessment of the PainRELife mobile app using the Mobile Application Rating Scale (MARS) [37,38] (n=23).

Behavior change assessment	Response, n (%)				
	Strongly disagree	Disagree	Undecided	Agree	Strongly agree
Awareness^a^	0	0	1 (4)	3 (13)	19 (83)
Knowledge^b^	0	0	0	7 (30)	16 (70)
Attitudes^c^	0	1 (4)	6 (26)	9 (39)	7 (30)
Help seeking^d^	0	3 (13)	6 (26)	5 (22)	9 (39)
Intention to change^e^	0	2 (8)	7 (30)	5 (22)	9 (39)
Behavior change^f^	4 (17)	2 (9)	9 (39)	3 (13)	5 (22)

^a^“This app is likely to increase awareness of the importance of addressing chronic pain.”

^b^“This app is likely to increase knowledge/understanding of chronic pain.”

^c^“This app is likely to change attitudes toward improve chronic pain.”

^d^“This app is likely to increase intentions/motivation to address chronic pain.“

^e^”Use of this app is likely to encourage further help-seeking for chronic pain.”

^f^“Use of this app is likely to decrease chronic pain.”

### Frequency of Use and Pain Self-Efficacy

According to the Student *t* test, younger participants used the mobile app less (mean 44.15, SD 7.11) than older participants (mean 50.33, SD 8.80; t_23_=–1.937, *P*=.03; *d*=0.77). A difference in pain self-efficacy was observed between participants with higher versus lower frequency use (t_23_=1.644, *P*=.057; *d*=0.65). The latter data indicate that, at T2, participants with a lower pain self-efficacy (mean 40.83, SD 14.58) used the mobile app more than participants with a higher pain self-efficacy (mean 48.46, SD 7.90).

### Pain Intensity and Shared Decision-Making

The repeated measures ANOVA revealed that participants reported a reduction in pain intensity from T0 (mean 5, SD 1.68) to T2 (mean 3.72, SD 2.59; *F*_2,48_=3.407, *P*=.04). A positive correlation was found between the total number of times the mobile app was accessed and pain intensity at T2 (*r*=0.458, *P*=.03).

No correlations were detected between the MARS subscales and PSEQ or NRS. A negative correlation was observed between the subjective quality subscale and the number of times the mobile app was accessed (*r*=–0.498, *P*=.02). Further, the engagement (*r*=0.445, *P*=.03), information (*r*=0.427, *P*=.04), and subjective quality (*r*=0.548, *P*≤.007) subscales were positively correlated with shared decision-making.

## Discussion

### Principal Findings

Considering the primary endpoint of this pilot study, patients with breast cancer provided a generally positive rating for the usability of the PainRELife mobile app*.* Patients with breast cancer appreciated both the quality and quantity of the health information provided on chronic pain and how chronic pain should be managed during the survivorship trajectory. Specifically, the information in the different sections and modules were perceived as informative and comprehensible (20/23, 87%) and from credible sources of information (18/23, 78%). Most of the patients with breast cancer reported that habitual use of the mobile app helped increase help-seeking behaviors for chronic pain (14/23, 61%), their general attitudes toward chronic pain, and their willingness to identify preeminent strategies for managing chronic pain. These results are particularly noteworthy considering that many studies have suggested that chronic pain syndrome in patients with breast cancer is commonly undiagnosed and not often considered by oncologists [8]. In addition, many cancer patients report poor knowledge about cancer-related chronic pain, available interventions, and possible health system resources [44].

Furthermore, the positive correlation between the total number of times the mobile app was accessed and pain intensity (*P*=.03) might indicate that patients with breast cancer who had a higher pain level might have utilized the mobile app to find evidence-based information and strategies to self-manage their pain. This possible interpretation might be linked to the difference in pain self-efficacy observed between the participants with higher versus lower frequency mobile app use. Specifically, participants with lower pain self-efficacy used the mobile app more than participants with higher pain self-efficacy. Perhaps the participants with lower pain self-efficacy used the mobile app to find a strategy or way to manage their pain. Self-efficacy has a crucial role for patients with cancer, and studies have reported that it improves psychological well-being, reduces fear of cancer recurrence, enhances self-care, and improves management of symptoms such as pain [45,46]. Vinnikova et al [47] observed that individuals might use mobile apps to learn more about their health problems and monitor their physical and psychological status. Furthermore, considering that self-efficacy is an attribute of cancer pain self-management, the prevalent use of the mobile app by participants with a lower self-efficacy could explain the percentage of participants who reported concerns about intention to change and the capacity to transform intention into a fundamental behavioral change [48].

A second series of results are linked to the secondary endpoints. Participants reported a reduction in pain intensity at 3 months (*P*=.04). We argue that the use of the mobile app might have relieved the pain experience, disease, and treatment-related symptomatology as observed in other previous studies [16,19]. One noteworthy finding is related to the association between specific features of the mobile app, evaluated with the MARS, and shared decision-making. Indeed, participants who provided higher positive evaluations about engagement (*P*=.03), information (*P*=.04), and subjective quality (*P*=.007) also reported higher perceptions of having shared decisions along their care pathway. We argue that patients with breast cancer who feel involved in their treatment decisions are more engaged with the mobile app. For this type of patient, information retrieved in the mobile app might be used to reinforce and reiterate the ability to achieve a shared decision throughout their care pathway.

### Limitations

Despite the interesting and challenging results, this pilot usability study had some limitations that must be considered. The primary limitation is the relatively small sample size of patients with breast cancer and the decision to use a single group to test usability. This decision might have caused a loss of pertinent information about the patients’ perceptions of the usefulness of this digital health technology. However, our sampling strategy is consistent with the pilot study design and methodological guidelines [49-51]. Related to this point, we must also mention that the statistical significance reported for pain self-efficacy might be considered borderline (*P*=.057). However, the effect size is medium-to-large (*d*=0.65), which supports the statistical difference between the groups. We argue that the *P* value might be due to the small sample size [52]. We also argue that this could be a significant result that has to be further investigated in successive studies, considering the positive effect of cancer pain self-management on QoL, when compared with pharmacological treatments such as opioid consumption [48].

In addition, the “frequency of use” variable had a moderately high SD (15.60). However, the distribution of the participants between the low frequency (n=13) and high frequency (n=12) groups was homogeneous and balanced. Furthermore, we hypothesized the presence of selection bias resulting from the inclusion criteria, which required participants to have internet access and a personal smartphone. This criterion may have limited the inclusion of certain vulnerable groups among patients with breast cancer, such as older adults or individuals with lower health literacy and socioeconomic challenges who could be at higher risk of undiagnosed chronic pain. Most of our patients with breast cancer also reported medium-to-low pain during the entire study and were in the acute and extended stages of the survivorship trajectory, which might have affected the frequency of mobile app use. Indeed, even if the total number of times the mobile app was accessed was relatively high and satisfactory, in the last month of the study, some participants decreased their total usage; 2 of 25 participants used the mobile app only at enrollment. The last limitation is related to the previous one and concerns the lack of assessment of the timing of mobile app use. Indeed, only the total number of times the mobile app was accessed was collected and evaluated. These limitations have been intensely discussed in the full protocol published elsewhere, and we plan to address them in future studies [7].

### Conclusions

The data retrieved from this pilot study evaluating patients’ experiences using a novel and integrated health ecosystem for the management of chronic pain for breast cancer survivors are consistent with other studies highlighting that digital health technologies, when developed using a patient-driven approach, might be considered valuable tools for increasing the participation of patients with breast cancer in clinical care. In addition, these tools permit the achievement of critical clinical outcomes and improvement in QoL [4,8,22]. Moreover, health-integrated ecosystems permit secondary key outcomes such as reducing the burden on health care professionals and optimizing health system resources. Finally, we argue that digital integrated health ecosystems might be important devices for improving the ongoing monitoring of physical status, psychological burden, and socioeconomic issues during the cancer survivorship trajectory.

## Abbreviations

ASyMS: Advanced Symptom Management System
FHIR: Fast Healthcare Interoperability Resources
IEO: European Institute of Oncology
MARS: Mobile Application Rating Scale
NRS: numeric rating scale
PECAN: Pain Education after Cancer Collaborative
PSEQ: Pain Self-Efficacy Questionnaire
QoL: quality of life
SDM-Q-9: 9-item Shared Decision-Making Questionnaire

## Data Availability

The data sets generated and analyzed during this study are available from the corresponding author upon reasonable request.
